# Evil of Smoking Tobacco

**Published:** 1859-08

**Authors:** 


### EVIL OF SMOKING TOBACCO, AND ITS NATIONAL COST.

A controversy is just now going on in Glasgow, between Mr. William Logan and Dr. M'Leod, as to the utility or the evil of tobacco-smoking. Mr. Logan uses some very forcible argument against the employment of the "weed." He says he had lived—

"In London, Leeds, Rochdale, Bradford, and Glasgow, for upwards of sixteen years amongst the humbler classes; and whilst he had met with thousands of inveterate smokers, he never found one of them attempt to defend smoking, but they almost invariably referred to it, of their own accord, as a 'bad habit,' and regretted that they had been foolish enough to learn it. The only occasion on which he had seen tobacco used with apparent advantage was when visiting, some eighteen months ago, the inmates of the Lunatic Asylum at Edinburgh, where the intelligent medical superintendent gratified about a dozen of the unfortunate inmates by quietly dividing amongst them about half an ounce of tobacco."

